# A community–based system dynamics approach for understanding factors affecting mental Health and Health seeking behaviors in Beirut and Beqaa regions of Lebanon

**DOI:** 10.1186/s12992-020-00556-5

**Published:** 2020-03-30

**Authors:** Aya Noubani, Karin Diaconu, Lilian Ghandour, Maria El Koussa, Giulia Loffreda, Shadi Saleh

**Affiliations:** 1 Global Health Institute, American University of Beirut, Beirut, Lebanon; 2 NIHR Research Unit on Health in Situations of Fragility, Institute for Global Health and Development, Queen Margaret University, Musselburgh, UK; 3 Department of Epidemiology and Population Health, Faculty of Health Sciences, American University of Beirut, Beirut, Lebanon

**Keywords:** Mental health, Health seeking, Syrian refugees, Lebanese community, Fragile context

## Abstract

**Background:**

Available evidence on mental health and psychosocial problems in Lebanon is limited. Recent quantitative data suggests a high prevalence among Syrian refugees and their Lebanese host communities, with significant treatment gaps in both populations. This study aims to determine how Lebanese host and Syrian refugee communities perceive mental health, and identify health seeking behaviors and barriers to health access in two contrasting contexts of fragility.

**Methods:**

A comparative qualitative study design was adopted whereby a total of 36 semi-structured interviews with Lebanese host and Syrian refugees’ community members were conducted, followed by a series of four participatory group model building (GMB) sessions. Participants were recruited from two contrasting fragility contexts: Beirut and Beqaa regions. During these sessions, causal loop diagrams were elicited depicting shared understandings of factors prompting the onset of mental health and psychosocial issues; health seeking behaviors, pathways and elements affecting the rate of health improvement and maintenance were also identified.

**Results:**

Community members in both settings had similar perceptions of factors contributing to mental health. Participants named long-term effects of exposure to wars, political and social effects of conflicts, and financial constraints at the household level as precipitating factors prompting the onset of mental health and psychosocial stressors. Gender and integration related challenges between communities were identified as factors that affect condition onset and associated care seeking. Pathways for health seeking were found to be shaped by trust, the advice and support of loved ones, and the need to ensure confidentiality of affected individuals. Recurrent themes in discussion highlighted major barriers to healthcare access including significant delays in health care seeking from the formal health system, widespread social stigma, prohibitive service costs, lack of health coverage, limited awareness of mental health service availability and limited trust in the quality of services available.

**Conclusion:**

Mental health and psychosocial support strategies need to be gender- and integration-sensitive, primarily focused on condition prevention and awareness raising in order to strengthen health-seeking behaviors.

## Background

Mental illness, defined by the World Health Organization (WHO) as a group of disorders “characterized by some combination of abnormal thoughts, emotions, behaviors, and relationships with others” [1], is a rising global health challenge affecting a broad range of individuals across all age-groups [1]. Mental and substance abuse disorders were found to be associated with 13% of global Disability-Adjusted Life Years (DALYs) and 32% of Years Lived with a Disability (YLDs) worldwide [2]. In addition, individuals with these disorders were found to face increased rates of morbidity from general medical conditions [2–5]. Nevertheless, individuals with mental illnesses can lead productive and fulfilling lives given appropriate medical and nonmedical management [6, 7].

There are disparities in outcome and quality of care among people with mental health disorders, with less favorable outcomes seen in ethnic and racial minorities [8] and those from lower socio-economic status groups [9]. Despite advances in behavioral and pharmacological therapies, it is estimated that a disproportionate number of affected individuals will remain untreated - especially in low and middle-income countries (LMICs) [10]. These countries often have limited healthcare resources – and it has been estimated that over 75% of mentally ill individuals go untreated due to a shortage of mental health services [10, 11]. While approximately 50% of individuals suffering from a mental health problem seek treatment in high income settings, this is far more than the scant 10% who are estimated to seek care in the developing world [10]. Despite the contribution of mental health disorders to the global burden of the disease, the quality of care received remains suboptimal, and there are persistent gaps in access to, and receipt of, mental health services worldwide and specifically in LMICs [12].

### The Lebanese setting

Lebanon is a low-middle income country in the Eastern end of the Mediterranean Sea bordered by the Syrian Arab Republic and the occupied Palestinian territory. The burden of mental health disorders in the Middle East has been exacerbated by the growing problems associated with numerous long-standing armed conflicts [13]. Over the years, Lebanon has endured repeated shocks to its health care system; governance and financing challenges have been exacerbated due to political instability, and the displacement of Syrian refugees into the country has placed additional pressure on the system [14]. Since 2011, the Syrian crisis has resulted in more displacement than any other event in history [15]. Lebanon has witnessed a 30% rise in its total population making it the host country with the highest number of displaced Syrian refugees per capita [16–18]. At this time, there are over 930,000 registered Syrian refugees on Lebanese grounds [19]. This demographic shift has had a considerable impact on the country’s health system, economy, employment and infrastructure [18]. The Syrian crises has also strained Syrian-Lebanese relations. In a recent survey, a minority of the of Lebanese (26.7%) ranked the quality of relations between Syrians and Lebanese as “very positive” or “positive”, compared to a larger proportion (42.1%) of the Syrian refugees [20]. As the Syrian crisis enters its ninth year, the host community fatigue with the protracted presence of Syrian refugees remains prevalent, mainly driven by competition over jobs and too much strain on Lebanon’s resources and services [20].

While displacement and exposure to trauma through conflicts and wars have been linked to an increased risk of mental disorders, only a small number of studies have examined these associations at the national level [21]. A prospective wartime study in four Lebanese communities identified a relatively high prevalence of post-traumatic stress disorders (PTSD) (29.3%) particularly in areas exposed to repetitive conflicts [22]. Mental health disorders in Lebanon are common, with an estimated 1 in 4 of Lebanese residents having had one or more mental health disorder in their lifetime; still, treatment-seeking is slow with a median time ranging from 6 to 28 years depending on the disorder [13]. The treatment gap is also evident in children and adolescents with mental health disorders where only 6% had sought treatment for their condition [14].

Absence of adult males among Syrian refugee populations exacerbates the vulnerability of Syrian children and women, who are subject to risks of child marriage, child labor, and sexual violence, all of which can lead to mental health problems [23]. The predominance of adolescents and children among Syrian refugees is likely explained by the fact that men remained in Syria to protect their businesses or houses. In 2016, a vulnerability assessment survey estimated that 3% of households reported one or more residents in need of mental care [24], but only 38% of respondents reported having sought professional treatment. Difficulties faced in accessing treatment services included cost of treatment, consultation fees, rejection from a facility or simply not knowing where to seek help [24]. Alsharabati et al.*,* (2015) found that 37% of the Syrian refugees in Lebanon reported worse treatment than others (i.e. Lebanese, foreign individuals) when seeking access to services including health related ones [25]. To compound this, over a third of Syrian refugees stated that amongst all offered services, health-related ones were the most difficult to access [24].

While Syrian refugees are given free access to primary healthcare services provided by humanitarian international organizations, local NGOs, the Ministry of Public Health (MoPH) and the Ministry of Social Affairs [23], these services are unable to accommodate the rising health needs. Despite the UNHCR covering some healthcare costs such as hospitalization, a high number of refugees are still unable to afford the remaining costs [23]. Additionally, services are generally limited to refugees registered with the UNHCR; unregistered refugees have limited recourse to healthcare support [23].

### Rationale

Given high levels of mental distress, their underlying factors, and the high treatment gap encountered in Lebanon, it is critical to understand the determinants behind delays in health care seeking. More specifically, it is important to further understand the constraints that residents of Lebanon – both host communities and Syrian refugees – face when attempting to access mental health services.

This study adds to the published literature by examining how Lebanese host and Syrian refugee communities in two contrasting settings perceive mental health and articulate mental health problems, and further, by investigating the dynamics of whether and how these populations access treatment services, and the main challenges faced by users in their health seeking and treatment journey. The findings will help inform Lebanon’s national mental health strategy and its aims.

## Methods

### Research design

We adopted a qualitative comparative design to examine the factors and dynamics affecting mental health and psychosocial support (MHPSS) onset and associated health-seeking behaviors. The study consisted of semi-structured interviews and four Group Model Building (GMB) workshops. The study was conducted in two contrasting contexts of fragility in Lebanon: Beirut and Beqaa, and targeted two population groups – Lebanese and Syrian refugees. Given that discussions on the topics of interest are likely to be shaped by participants’ gender, we split GMB participants into gender specific groups to address any sensitivities [26–28].

### Study setting

The two regions Beirut and Beqaa were purposively chosen to reflect settings with a diverse fragility profile [29]. A scoping review on health and fragility – suggests that ‘fragility’ arises where links between the community and health systems are most disrupted. This can be due to historical circumstances (e.g. previous conflict), difficulties in service delivery (e.g. as when services are overwhelmed by influx of displaced populations), community living conditions (e.g. as in crowded, urban and poor environments) or any combination of the above [30]. Beirut is the capital city and the main urban center of the country and Beqaa is a largely rural region greatly impacted by the Syrian refugee influx. Beqaa hosts a larger percentage of the Syrian refugees (36%) compared to Beirut (24%) and according to the UN-Lebanon Interagency taskforce, the Beqaa region requires major health institutional support [19]. Second, poverty rates are generally estimated to be the lowest in the Greater Beirut area (between 16 and 22%) and the highest in the Beqaa governorate (38%) [31]. The economic situation in Beirut attracts wealthier Syrian refugees; by contrast, in the Beqaa region most refugees live in tented settlements [32]. The Beqaa region is characterized by poor developed infrastructure, remote areas, and a relatively weak local civil society structure. This region has been hit hard with the reduced economic border trade with Syria, deteriorating security in many areas, and the enormous pressure of Syrian refugees on the host communities, which contributed to inadequate provision of social services for refugees [32].].

### Study population, sampling and recruitment

The study included a diverse gender- and age-balanced group of both Syrian refugees and Lebanese host community members. General community members (adults aged over 18) as well as caretakers of people affected by MHPSS issues (e.g. parents of children aged 10–18) were eligible to participate. Purposive, convenience and snowball sampling techniques were all used to recruit participants. Women, men and parents meeting the eligibility criteria detailed above were purposively sampled.

Non-Governmental Organizations (NGOs) active in Greater Beirut and Beqaa offering MHPSS services were contacted and asked to assist in the recruitment of community members meeting the target participant profile. If they agreed, facility managers were asked to share the consent form with potential participants. Primary health care centers in the two regions were visited to recruit participants after receiving the approval from the director of the center.

In addition, Syrian refugees were recruited through the list of registered Syrians (via UNHCR); the latter provided our research team with the surnames and phone numbers of registered Syrian refugees for our research team to make contact. Identified participants were contacted via phone by a trained researcher who provided a brief explanation of the study; once informed oral consent was obtained and participants were recruited into the study.

Based on the availability of participants, they were asked to participate either in the GMB workshops or semi- structured interviews. Participants were also asked whether they could recommend any persons that met our target participant profile; if so, these persons were also approached.

Overall, we recruited a total of 89 participants from both contexts and communities (Table 1).

**Table 1:** Participant characteristics by location, gender and data collection modality

Targeted participant group	Data collection modality	Beirut	Beqaa
Lebanese community	GMB workshops (*N* = 2)	16 Total 9 Females 7 Males	12 Total 9 Females 3 Males
	Semi structured interviews (*N* = 18)	9 Total 5 Females 4 Males	9 Total 6 Females 3 Males
Syrian Refugees	GMB workshops (*N* = 2)	16 Total 10 Females 6 Males	9 Total 2 Females 7 Males
	Semi structured interviews (*N* = 18)	9 Total 5 Females 4 Males	9 Total 5 Females 4 Males

#### Semi-structured interviews: data collection and analysis

We conducted 9 interviews with women, men and parents across each setting respectively and per community group (Syrian and Lebanese); a total 36 interviews were carried out. All interviews were conducted in Arabic, audio-recorded, and lasted on average 15 min. The setting for interviews was decided based on the preference of the participant; they were conducted at either the health center, households or refugee’s tents. Interviews were semi-structured and included predetermined, open-ended questions on:

- Definitions of mental health and the causes contributing to mental health issues
- Society’s perception of those affected by mental health problems
- Help seeking behaviors and factors shaping these routes
- Perceptions of treatment-seeking from the health system and its barriers

To identify the general themes to be discussed in the GMB workshops, the research team met and preliminarily analyzed a sample of interview data to identify prevalent themes to be explored in the workshops. Post-workshops, all interviews underwent thematic analysis. Two researchers read the transcribed interviews in order to familiarize themselves with the information provided; these researchers then open-coded a sample of transcripts and in discussion refined a coding framework. Researchers further compared coding practice via an inter-rater reliability test (Kappa statistic = 0.8). Following this, all transcripts were independently coded, then underwent review by the group, with themes and sub-themes emerging in an iterative process. Researchers then met to discuss emergent findings, and as necessary, case-sensitive analysis was also conducted to account for similarities and differences between the Lebanese and Syrian communities, male and female participants as well as participants from Beqaa and Beirut. Analyses were conducted using Dedoose [33].

#### Group model building: data collection and analysis

GMB is a methodological tool used by systems dynamics researchers, which allows researchers and relevant health stakeholders (e.g., patients, providers, interest groups) to come together and, in a participatory manner, elaborate conceptual models of system behaviors/problems under study [34]. Within this study, each GMB workshop was attended by around 15 persons with an active interest in MHPSS, or persons who act as caregivers of individuals affected by a mental health condition. GMBs were held at the American University of Beirut (AUB), and transportation to/from AUB was arranged for all participants.

Group model building sessions are organized around a series of scripts that correspond to sequential activities carried out by the participants [34]. Scripts detailing each activity were elaborated and used by all workshop facilitators in order to ensure consistency in prompts given, and activities undertaken by the diverse participant groups. Scripts were adapted from Scriptopedia [35] and are available in Additional file 1: Appendix 1.

Each GMB session consisted of three sections. First, to start discussions on key factors that contribute or lead to the onset of mental health issues, and associated health seeking behaviors, participants were asked to draw rich pictures [36] that help in identifying key issues affecting mental health and the journey of health seeking behaviors. Second, participants were asked to develop graphs and describe trends depicting the mental health situation including the change in the prevalence of mental health, knowledge of mental health issues and awareness of the availability of MHPSS services among the populations in Lebanon over time. Third, participants were asked to review the rich pictures and graphs developed and abstract a key set of variables relating to the onset of mental health issues, health seeking and individual wellbeing. Using these variables, researchers assisted participants to build a preliminary concept model describing the dynamics of mental health condition onset, health seeking behaviors and experiences of securing individual wellbeing. Fourth, participants were prompted to identify particular points of fragility (i.e. areas of particular weakness) and also intervention (i.e. areas where intervention would be most strategic) in the depicted concept models.

Connections between the variables as described by participants in their initial concept models were translated into an electronic model using the bespoke software VenSim [37]. Similar to interviews, GMB models underwent iterative analyses: first, using notes from the GMB sessions, variables and pathways in the concept models were refined and as needed consolidated to ensure the concept models reflected in the underlying causal logic of participants.

The resulting causal loop diagrams then underwent further iterative critical analyses. Specifically, the team compared models developed across the different workshops and groups therein (Beirut/Beqaa, host/refugee and male/female) and further consolidated information from these models into one overarching causal loop diagram. This entailed comparing variables and their definitions, comparing pathways to ensure both consistent and divergent information is captured and highlighting pathways and variables specific to only one group of participants. Balancing and reinforcing loops were then identified in this final causal loop model. Further, researchers discussed the leverage points affecting system behavior and compared these to the points identified by participants originally as fragility/intervention points. Associated interventions, as elaborated by participants, were then noted alongside the causal loop diagram according to the variables affected.

### Reporting

The reporting of this study followed the consolidated Criteria for Reporting Qualitative Research (COREQ) Additional file 1: Appendix 2.

### Ethics

Ethical approval was granted by the Institutional Review Board at the American University of Beirut (AUB) and Queen Margaret University (QMU).

## Results

We integrated findings from the interviews and the causal loop models and present these in relation to three main themes: 1) perceptions of mental health problems, 2) causes of mental health issues including relevant gender differences, and 3) health seeking behaviors and practices to maintain wellbeing.

### Perceptions of mental Health problems

Overall, participants from different settings and nationalities agreed that individuals with mental health issues are severely stigmatized. When asked about how people with mental health issues, like depression, are viewed by others, participants noted that mental health is not viewed or accepted as a disease that needs treatment but rather as a failure on the individual’s part to function normally or fit in.

*“In today’s society, if someone is struggling with mental health, people point fingers and say he is crazy.” A Lebanese man living in Beirut.*

*“They don’t view them positively. They would consider them crazy and as if they are less than human.” A Syrian woman residing in Beirut.*

Participants acknowledged that stigma is largely fueled by a lack of knowledge and information among communities.

*“There is no awareness. Some people think it’s because a person is not religious. They don’t take it like it’s a disease that needs treatment.” A Lebanese woman living in Beqaa.*

However, most of the participants mentioned that, in principle, they disagree with social stigma and discriminatory behavior. Similarly, participants emphasized that empathy plays a major role in making the life of people struggling with psychological issues easier. Moreover, many of them discussed the importance of seeking help from a therapist and normalized it. Only a minority of participants mentioned that the community is now more accepting of mental health issues and that the latter are becoming less of a social taboo.

*“It used to be a taboo issue and an embarrassing one. Now, people deal with it and accept it.” A Syrian woman residing in Beirut.*

### Causes of mental Health issues (refer to Fig. 1)

**Fig. 1:**
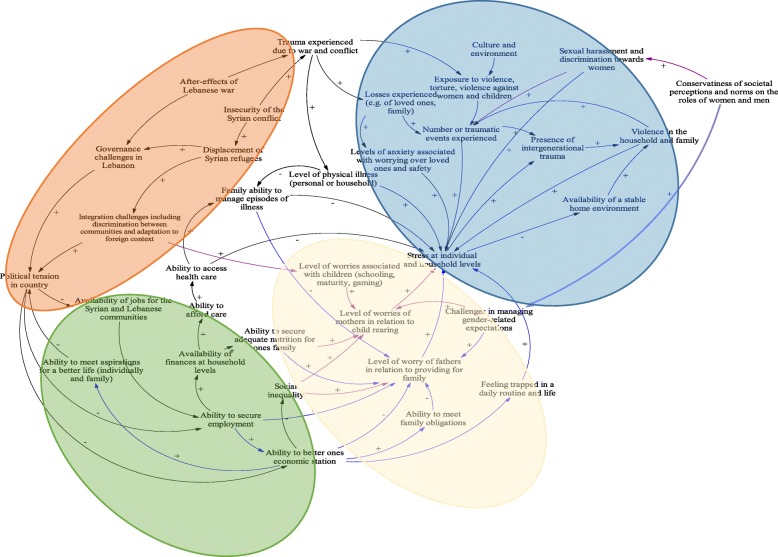
Factors affecting mental health and wellbeing

We proceeded to discuss findings corresponding to causes of mental health issues via reference to the causal loop model elicited via group model building sessions (Fig. 1). We distinguished four main categories of risk factors as identified by participants and discuss these below via reference to the causal loop diagram in Fig. 1.

#### Long term effects of exposure to war and violence (blue zone in Fig. 1)

The elaborated graphs and rich pictures (see Additional file 1: Appendix 3) identified that the Lebanese civil war as well as the Syrian crisis have both resulted in long-term effects on the mental health wellbeing of both communities. Participants talked about the loss of loved ones, families, and homes as well as their exposure to violence. These events have led to constant worry over their own safety and that of loved ones, which is a main contributor to stress and mental health issues. Interview findings support these accounts:

“*When someone dear dies, this could also lead to mental illness.” A Lebanese woman from Beirut.*

*“Yes, people developed fear. If something explodes, they get afraid. They have psychological issues.” A Syrian woman from Beqaa.*

Participants also discussed the presence of interpersonal violence within families (upper right end of the blue zone), though such discussions were more common among Lebanese residing in the Beqaa area and among Syrian refugees in both contexts. Participants in these groups mentioned that the culture and environment shaped men to be aggressive and violent within their families and larger community. Female participants echoed this and spoke about episodic exposure to violence both in women and children. Consequently, both male and female participants from the Syrian communities and the Lebanese living in the Beqaa noted that children fall in the cycle of intergenerational trauma and repeat patterns of aggressive behaviors. Participants described that violence in the household and family, as well as sexual harassment towards women (noted in violet in the model), are major contributors to stress and mental health issues.

#### The political and social effects of war (Orange zone in Fig. 1)

When asked to discuss the trends and precipitating factors driving the mental health burden over time, participants talked about the impact of wars and conflict on political stability and the socioeconomic situation of the population in Lebanon. Participants identified the Lebanese civil war as well as the Syrian crisis as key events that kept the Lebanese political system vulnerable to corruption and over time precipitated the erosion of confidence in current governance (orange zone, upper left section of Fig. 1).

*“We are in a country where there is no stability. We don’t feel if our future is here or no. I have 2 kids; I always worry about whether I should leave this country or stay. Over 10 years, the situation has worsened. I never used to think of leaving the country, but now I feel that I have to if I want my kids to grow up in good conditions.” A Lebanese man from Beirut.*

Lebanese participants described the influx of Syrian refugees to Lebanon as considerable and attributed the aggravation of the already existing local challenges in governance and the economy to this influx (see next section for how these challenges affect socio-economic fabric of Lebanon). Relatedly, participants from both communities mentioned that the displacement had led to integration challenges and this in turn exacerbated social tension in the country. One frequently mentioned example included discriminatory practices in schooling. Separate schooling times for Syrian and Lebanese children are necessary to manage demands given the scarce national resources (and different educational systems); however, this means children mix rarely and teachers are overworked and thus resort to discriminatory practices towards Syrian children.

#### Socioeconomic constraints (Green zone in Fig. 1)

As noted above, participants described political instability and governance challenges in the country as major factors impairing economic growth. Participants from both communities and residing in the two contexts talked about issues of unemployment and expensive livelihoods (Green zone, lower left part of the model in Fig. 1). The inability to secure employment impeded the improvement of an individual’s economic status and rendered them unable to afford daily life needs for their families, including children, as well as access to affordable healthcare in case of illness.

Syrian refugees noted that the loss of their properties/households and displacement to Lebanon has led to their struggle with harsh financial situations. The rules and regulations followed by the Lebanese government, starting with the legal restrictions on employment and the lack of appropriate aid and resources, have added to the stress and worsened their financial situation. Consequently, many Syrians were looking for informal jobs but were facing discrimination regarding their salaries.

*“If you go looking for a job, they barely pay you 200 or 250 ($). You work so hard, and you barely earn anything. You feel your efforts are in vain. All this affects how you feel. Yet, I hope we remain healthy and capable of working.” A Syrian man living in Beirut.*

Within the Lebanese participants, most mentioned that they are competing with Syrian refugees in the labor market, which is resulting in the host communities’ decreased ability to secure employment. Lebanese participants stated that Lebanese employers prefer Syrian refugees over locals because of their acceptance of lower salaries; while official employment of Syrians is highly regulated, regulation relating to salary scales of Syrians is lacking.

#### Gendered expectations driving onset of mental health issues (yellow zone in Fig. 1)

We noted gender differences for the drivers of stress and mental health issues within the family. Men considered financial obstacles as major drivers to stress and mental health issues (demonstrated by the blue arrows) because of their failure to secure employment, improve their family’s economic situation and meet aspirations for a better life.

*“There are many causes. I feel men worry more about financial matters, if he is unable to make his family happy. Men feel that money is the most important thing that they can offer to make their family happy. Mothers worry about taking care of their children, if they are not in a good mental state, if they have marital problems, or if they can’t meet their children’s demands. This causes stress. Children need to be well educated.” A Syrian woman living in Beirut.*

*“First, poverty can lead to a mental illness. When someone has no money and is unable to make ends meet or provide for his children, this greatly affects a person’s mental state. Just imagine not being able to secure the needs of your children, such as education, food, cloths. This has a major effect on parents’ mental state. This also affects children who would have nothing.” A Lebanese man living in Beqaa.*

Women from both communities described their worries in a different way (purple arrows). They highlighted the challenges of social inequality and the patriarchal social system whereby men predominate in certain roles within the job market or within the households. Women described their tasks of child rearing and household responsibilities as overwhelming and stressful. Syrian woman, specifically, talked about their constant worry about their children who face lots of discrimination and bullying at schools in Lebanon.

### Health seeking behaviors and practices to maintain wellbeing (refer to Fig. 2)

Participants of both communities and areas reflected on a consistent set of health seeking behaviors. We elaborate further on the factors affecting the patterns of health seeking, the diverse routes of health seeking, and drivers and barriers for seeking support from the health system (variables highlighted in green).

**Fig. 2:**
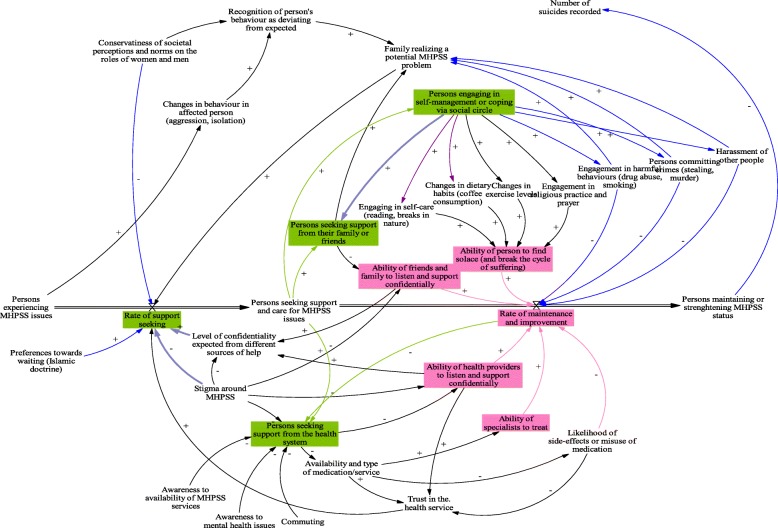
Health seeking behaviours and Practices to maintain wellbeing

The level of confidentiality expected from different sources of help, as well as the stigma around mental health issues, were the main factors shaping the rate of seeking support as mentioned by participants from both genders and communities (violet colored arrows).

#### Determinants of health seeking (green pathways of Fig. 2)

Health seeking behaviors are determined by the social connectedness of the persons experiencing mental health issues. Participants considered their families, friends or partners as the frontlines for support, because the latter are able to listen and provide support confidentially. However, gender differences were apparent. Women noted they were more likely to open up to family and friends as they are the main source of support for people suffering from mental health issues. Women explained that one seeks support from the people who can be trusted, who are able to listen actively and to provide support for the individual. However, men explained that they are less likely to talk about their issues and would keep them to themselves. Men further expressed a preference towards waiting before seeking support; in adherence with their religious doctrines, they considered that God is testing their patience when passing through life hardships and struggles.

A minority group of both genders mentioned that they will not disclose to anyone because they do not want to burden their loved ones. These participants in particular noted they would engage in self-management and diverse coping mechanisms in order to find solace and break the cycle of suffering. Different self-coping mechanisms were mentioned by women and men, such that women tend to engage in self-care and changes in dietary habits (violet/ purple arrows); men mentioned that a person would resort to harmful habits and behaviors such as smoking, alcohol and in dire circumstances even resorted to committing crimes (blue arrows).

When the social network and the coping mechanisms fail to secure the wellbeing of the affected person, seeking support from the health system is viable (highlighted in green). Participants mentioned that the substantial delay in accessing the health system is principally due to social stigma.

*"I: What are the obstacles that stand in the way of people going to a therapist?*

*“They would think that they’d seem crazy.” A Syrian man living in Beqaa.*

*“Our society views you as crazy if you see a therapist. So, society stands in your way when you want to seek therapy even if it is the only thing that would help you”. A Syrian man living in Beirut.*

#### Barriers to health seeking (variables highlighted in pink of Fig. 2)

Participants talked about the lack of trust in the ability of the health care system to improve and maintain the condition of patients. Particularly, participants noted limited trust in the quality of services being delivered and in the ability of the specialists to treat MHPSS cases (pathways and influences highlighted in pink). All participants mentioned that patients doubt the ability of the healthcare provider to listen and support confidentially, and they lack trust in the ability of the provider to treat and prescribe the appropriate medications. Lack of trust in both these processes, as well as the variable perceived quality of such services, were noted to affect the rate of wellbeing maintenance and improvement (variables highlighted in pink, lower right end of the model).

*“Some people get scared. They tell you they don’t want to go because their condition might worsen. They want to solve their problems alone. If society is not helping, he can go to the doctor, but he does not want anyone to intervene.” A Syrian woman living in Beqaa.*

In interviews, some people explained that it is not only trust in the system overall that is compromised but also in specific services provided.

*"Why wouldn’t some people accept that?*

*“Some people don’t trust in therapy. They don’t think it is effective.” A Lebanese man living in Beqaa.*

The majority of participants mentioned that the level of awareness of mental health issues and the willingness to improve could play a major role for seeking health and therapy (lower right part of the model). Moreover, participants residing in Beqaa mentioned the lack of available services and the long commute as major barriers to seeking help from the system (lower right part of the model).

*“Another factor is the area of residence, for example if I want to be treated by a good doctor but I live far from his clinic and I cannot always go to him. These are also minor obstacles.” A Lebanese woman living in Beqaa.*

*“ Sometimes these services would not be available at a center nearby or if no one is supporting the person with a mental illness and encouraging him to get treatment.” A Lebanese man living in Beqaa.*

Financial barriers and the high costs of treatment were also mentioned as major challenges to healthcare access, particularly in the Beqaa.

*“Sometimes it is expensive, so the cost stands in the way. Not everyone can afford it.” A Lebanese woman living in Beqaa.*

*“Mostly Financial obstacles. Unless there is social security or insurance” A Lebanese man living in Beqaa.*

### Points of fragility and suggested interventions

Participants were asked to reflect on the whole causal loop diagrams they developed and identify and vote for the top problem areas (so called points of fragility) affecting their mental well-being and health seeking journey (see Additional file 1: Table 1 in Appendix 4). Participants were then asked to identify interventions actively targeting these areas (See Additional file 1: Table 2 in Appendix 4).

Participants across both settings and communities identified the precarious financial situation at the household level, as influenced by unemployment, as a major challenge to mental health and wellbeing. The Lebanese community in particular highlighted that the political instabilities and corruption in Lebanon were major reasons for poverty, and were obstacles for development and prosperity.

At the individual level, women highlighted that family issues and the many expectations associated with raising children were factors that increased stress and precipitated the worsening of ones’ mental health problems. In contrast, men were mainly focused on employment and the socioeconomic situation in the country.

Towards the end of each workshop, participants were given the opportunity to suggest solutions and potential interventions to address the points of fragility in the system that they had highlighted. The solutions offered were focused on improving socioeconomic conditions, enhancing mental health awareness and service utilization as well as improving integration and acceptance of Syrian refugees in the host community. All participants noted the need for job opportunities with better salaries, which would enhance their financial situation and living conditions, and ultimately improve their psychological well- being. Moreover, Lebanese participants highlighted the need for more awareness campaigns and strategies to fight the widespread stigma around mental health issues, and the importance of reducing the costs of mental health treatment in Lebanon. They clarified that campaigns should aim to increase the level of knowledge on mental health issues, educate people on mechanisms for coping and prevention, as well as inform the community about the availability of services. In contrast, Syrian community members emphasized solutions revolving around addressing issues of discrimination and integration within Lebanese host communities, in order to improve the relations and reduce the social tension among both.

## Discussion

**Fig. 3:**
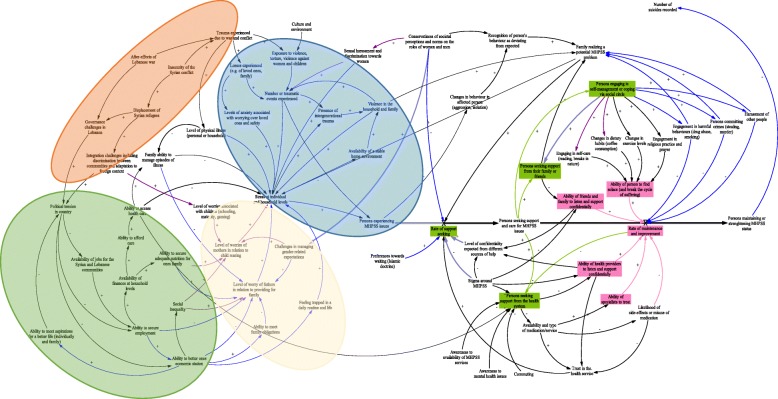
Causal Loop diagram showing causes affecting mental health and pathways for health seeking

This is the first study to elicit and interrelate a range of factors contributing to mental health wellbeing and health seeking behaviors as perceived by community members from the Syrian refugee and Lebanese host community in two fragile contexts in Lebanon (refer to Fig. 3). The participatory workshops provided means to instigate an open discussion on mental health issues and allowed community members from different nationalities and experiences, age groups and contexts to open up and talk about their perceptions on a socially stigmatized topic. The causal loop diagram that was developed acts as a helpful starting point to gain insight into the locally and contextually specific factors that influence mental health in this context (refer to Fig. 3). Personal issues (such as availability of finances at household levels) were related to wider structural determinants such as political and social effects of wars as well as the long-term effects of exposure to violence and conflict. Our findings highlight a relatively consistent set of health seeking behaviors intended to maintain and secure wellbeing, including approaching family and friends in the first instance, engaging in self-management and self-coping mechanisms, and ultimately seeking the health system’s services. Our community participants identified particularly fragile points which they prioritized for further strengthening, thus offering insight into the relative priorities of community members in relation to MHPSS services in Lebanon.

The results corroborate as well as extend the limited prior research done in Lebanon on mental health by providing a deeper understanding of the range and interconnectedness of causes leading to stress and mental health issues. Previous research has reported that socio cultural factors resulting from the internal and external wars witnessed in Lebanon were major contributors to mental disorders [21, 38]. Our findings were also in line with those reported on the factors that influence one’s psychological development and that extend to the realm of the home and family such as the financial constraints and violence against women and children [21, 39, 40]. To the best of our knowledge, this is one of the first studies reporting on the interconnected mechanisms of war effects on the integration challenges between Syrian refugees and Lebanese host communities. This includes discrimination and adaptation to the foreign context as perceived by the two parties. While UNHCR reported on the association between gender roles and MHPSS issues among Syrian refugees [41], no previous studies on the Lebanese community showed the association between gender-related elements and MHPSS issues.

Outcomes from this study shed light on the complexity of health seeking behaviors, especially when accessing the health system. A study by Karam et al., reported alarmingly low rates of help seeking among the Lebanese adult community shaped by tremendous delays between the onset of the disease and utilization of MHPSS services [21, 42]. Moreover, a study that aimed to study the prevalence of psychiatric disorders among Lebanese children and adolescents showed that only 6% of those with one or more mental health disorders had sought professional treatment [42]. Reported barriers to accessing mental health services in our study are comparable to those identified in the published literature. Stigma, lack of awareness about mental disorders, cost of treatment and trust in the quality of the services being delivered were major obstacles to accessing MHPSS care in Lebanon [24, 42].

### Strengths

The current study provides a contextually grounded account of the dynamics behind the onset of mental health stressors and health seeking journeys to maintain and secure mental health wellbeing in two contrasting fragile regions of Lebanon. Our findings add significant value to the limited evidence base in the region, particularly regarding Syrian refugees and local Lebanese host communities. Moreover, findings that emerged are formative and help generate new hypotheses, as well as identify potential areas for policy interventions. The causal loop diagram offers a lens to explore potential leverage points that may take the form of a policy, program, or intervention that strengthen the strategy of the National Mental Health Program (NMHP), which has been a critical steppingstone for the mental healthcare sector in Lebanon.

### Limitations

Given the limited sample of participants, our findings cannot be generalized and must be considered within their respective geographic and fragility contexts of Greater Beirut and Beqaa. We additionally acknowledge that our workshop participant group was biased towards participants from lower socio-economic groups; we attempted to mitigate this by interviewing persons of a diversity of backgrounds. Additionally, we acknowledge that researcher perspectives may influence the work and findings; researchers from diverse backgrounds collaborated on the synthesis of the causal loop diagram, and brought in diverse positions and perspectives in addition to that of the participants.

## Conclusion

Our model highlights important causal factors of MHPSS issues summarized by four main categories: the long term effects of exposure to war and violence, the political and social effects of war, socioeconomic constraints, and gender differences for the drivers of mental health issues within the family. The model also identified factors that prevent individuals with mental illness from accessing services. Our study demonstrates that group model building methods using community-based system dynamics may provide an effective tool to elicit a common vision on a complex problem; our analyses identified the need for gender- and integration-sensitive mental health and psychosocial support strategies, primarily focused on condition prevention and awareness raising in order to strengthen health seeking behavior.

## Supplementary information

**Additional file 1: Appendix 1.** The script of the GMBS. **Appendix 2.** Consolidated criteria for reporting qualitative studies (COREQ): 32-item checklist. **Appendix 3.** Rich pictures and graphs from the GMBs. **Appendix 4: Table S1.** Points of Fragility identified by the participants in the generated model. **Table S2.** Intervention suggestions by participants.

## Data Availability

All data generated or analyzed during this study are included in this published article [and its supplementary information files].

## Abbreviations

MHPSS: Mental health and Psychosocial support
GMBs: Group Model Building
WHO: World Health Organization
DALYS: Disability-Adjusted Life Years
YLDs: Years Lived with a Disability
LMICs: Low and middle-income countries
PTSD: Post-Traumatic Stress Disorders
NGOs: Non-Governmental Organizations
MoPH: Ministry of Public Health
AUB: American University of Beirut
QMU: Queen Margaret University
