# Supplementary material for: A community–based system dynamics approach for understanding factors affecting mental Health and Health seeking behaviors in Beirut and Beqaa regions of Lebanon
Source: Global Health. 2020 Mar 30;16:28. doi: 10.1186/s12992-020-00556-5 (PMC7106684; doi:10.1186/s12992-020-00556-5)
Supplement: Supplementary file 1 — Additional file 1: Appendix 1. The script of the GMBS. Appendix 2. Consolidated criteria for reporting qualitative studies (COREQ): 32-item checklist. Appendix 3. Rich pictures and graphs from the GMBs. Appendix 4: Table S1. Points of Fragility identified by the participants in the generated model. Table S2. Intervention suggestions by participants. [file 12992_2020_556_MOESM1_ESM.docx]

**Appendix 1: The script of the GMBS**

**Community level GMB**

| **Time** | **Activity** |
| --- | --- |
| (min) |  |
|  |  |
| 10 | Welcome and project introduction |
|  |  |
| 20 | Demonstrating use of GMB methods |
|  |  |
| 20-30 | **Rich picture** |
|  | The modeller’s prompt community members to think of a person with a MHPSS need/condition |
|  | within their community. Facilitators prompt the participants by using local idioms corresponding for |
|  | example to depression, anxiety, and epilepsy but encourage participants to reflect on wider MHPSS |
|  | conditions and name them. |
|  | What are the causes for these conditions/issues that prompt condition development? (5-10 minutes) |
|  | Can you draw a journey around these people? Where do they go to seek help and support? (5-10 |
|  | minutes) |
|  | Once drawings are elaborated, participants will be asked to feed back to the entire group. |
|  |  |
| 20-30 | **Variable elicitation: social connections** (Strang) |
|  | Participants will be encouraged to reflect on the previous scenarios – which are fairly |
|  | common either to their or their family member’s lives – and asked to “think of all the people or |
|  | organizations that someone in their community might talk to about such a problem or go to and ask |
|  | for help.” On a central flipchart, the facilitators will keep a note of the problem mentioned and the |
|  | list of people or organizations mentioned as relevant by community members. |
|  | Once all groups have completed their work, facilitators will compare elaborated lists and decide |
|  | whether to proceed to merge the list of all resources/keep separate resource lists. |
|  |  |
| 10-20 | **Break** |
|  |  |
| 30-40 | **Pathways of help-seeking** |
|  | During this exercise, facilitators will use the cards depicting the resource people/social connections |
|  | previously elicited and ask participants to draw a help seeking pathway. |
|  | Participants will be asked to reflect on where community members go first, why they go here and |
|  | additionally on what happens at this place/when connecting with this person. |
|  |  |
| 50-60 | **Break/lunch** |
|  |  |
| 50-60 | **Further elaboration of help-seeking models** |
|  | Based on the help seeking model developed above, facilitators will further prompt participants to |
|  | reflect on issues of trust, affordability and particular resource persons that may be mobilized to help |
|  | in preventive MHPSS service delivery. |
|  |  |

**Appendix 2: Consolidated criteria for reporting qualitative studies (COREQ): 32-item checklist**

Developed from:

Tong A, Sainsbury P, Craig J. Consolidated criteria for reporting qualitative research (COREQ): a 32-item checklist for interviews and focus groups. *International Journal for Quality in Health Care*. 2007. Volume 19, Number 6: pp. 349 – 357

| **No. Item** | **Guide questions/description** | **Reported on Page #** |
| --- | --- | --- |
| **Domain 1: Research team and reﬂexivity** |  |  |
| *Personal Characteristics* |  |  |
| 1. Inter viewer/facilitator | Which author/s conducted the interview or focus group? |  |
| 2. Credentials | What were the researcher’s credentials? E.g. PhD, MD |  |
| 3. Occupation | What was their occupation at the time of the study? |  |
| 4. Gender | Was the researcher male or female? |  |
| 5. Experience and training | What experience or training did the researcher have? |  |
| *Relationship with participants* |  |  |
| 6. Relationship established | Was a relationship established prior to study commencement? |  |
| 7. Participant knowledge of the interviewer | What did the participants know about the researcher? e.g. personal goals, reasons for doing the research |  |
| 8. Interviewer characteristics | What characteristics were reported about the inter viewer/facilitator? e.g. Bias, assumptions, reasons and interests in the research topic |  |
| **Domain 2: study design** |  |  |
| *Theoretical framework* |  |  |
| 9. Methodological orientation and Theory | What methodological orientation was stated to underpin the study? e.g. grounded theory, discourse analysis, ethnography, phenomenology, content analysis |  |
| *Participant selection* |  |  |
| 10. Sampling | How were participants selected? e.g. purposive, convenience, consecutive, snowball |  |
| 11. Method of approach | How were participants approached? e.g. face-to-face, telephone, mail, email |  |
| 12. Sample size | How many participants were in the study? |  |
| 13. Non-participation | How many people refused to participate or dropped out? Reasons? |  |
| *Setting* |  |  |
| 14. Setting of data collection | Where was the data collected? e.g. home, clinic, workplace |  |
| 15. Presence of non-participants | Was anyone else present besides the participants and researchers? |  |
| 16. Description of sample | What are the important characteristics of the sample? e.g. demographic data, date |  |
| *Data collection* |  |  |
| 17. Interview guide | Were questions, prompts, guides provided by the authors? Was it pilot tested? |  |
| 18. Repeat interviews | Were repeat inter views carried out? If yes, how many? |  |
| 19. Audio/visual recording | Did the research use audio or visual recording to collect the data? |  |
| 20. Field notes | Were ﬁeld notes made during and/or after the interview or focus group? |  |
| 21. Duration | What was the duration of the inter views or focus group? |  |
| 22. Data saturation | Was data saturation discussed? |  |
| 23. Transcripts returned | Were transcripts returned to participants for comment and/or correction? |  |
| **Domain 3: analysis and ﬁndings** |  |  |
| *Data analysis* |  |  |
| 24. Number of data coders | How many data coders coded the data? |  |
| 25. Description of the coding tree | Did authors provide a description of the coding tree? |  |
| 26. Derivation of themes | Were themes identiﬁed in advance or derived from the data? |  |
| 27. Software | What software, if applicable, was used to manage the data? |  |
| 28. Participant checking | Did participants provide feedback on the ﬁndings? |  |
| *Reporting* |  |  |
| 29. Quotations presented | Were participant quotations presented to illustrate the themes/ﬁndings? Was each quotation identiﬁed? e.g. participant number |  |
| 30. Data and ﬁndings consistent | Was there consistency between the data presented and the ﬁndings? |  |
| 31. Clarity of major themes | Were major themes clearly presented in the ﬁndings? |  |
| 32. Clarity of minor themes | Is there a description of diverse cases or discussion of minor themes? |  |

**Appendix 3: Rich pictures and graphs from the GMBs**


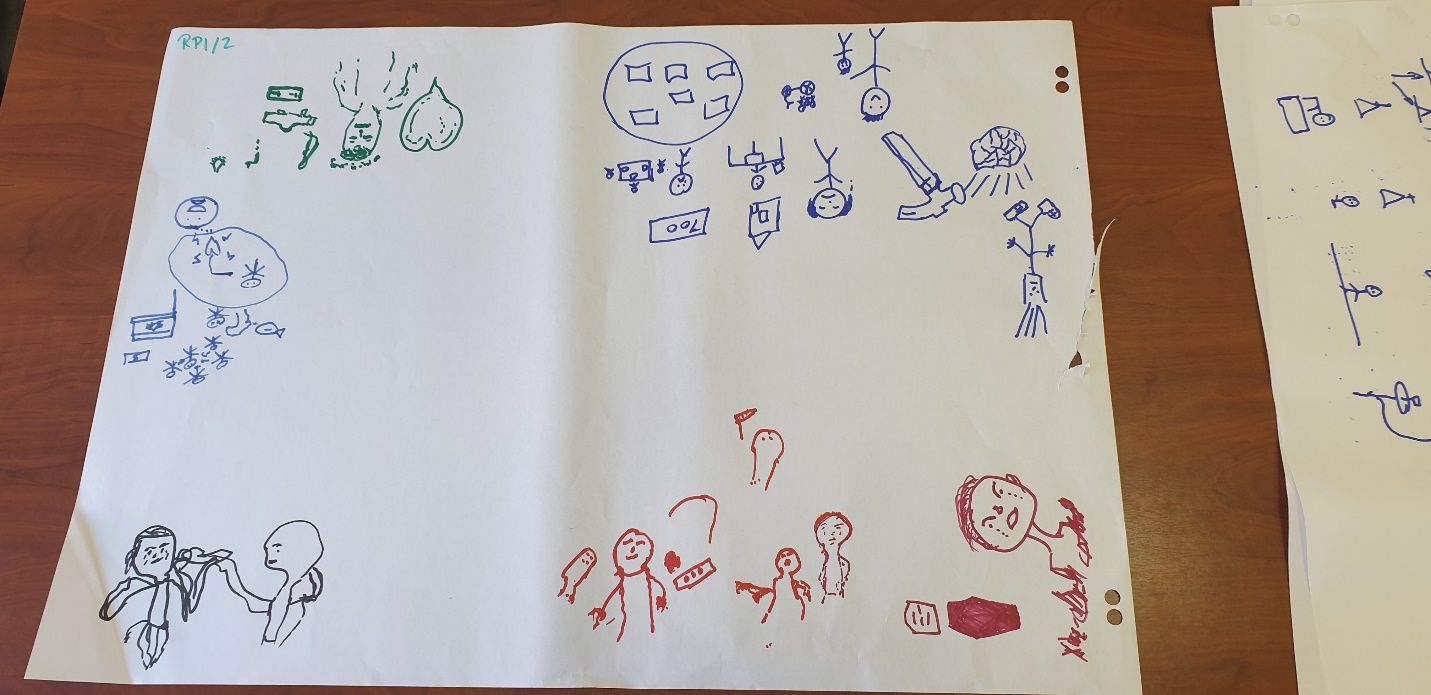


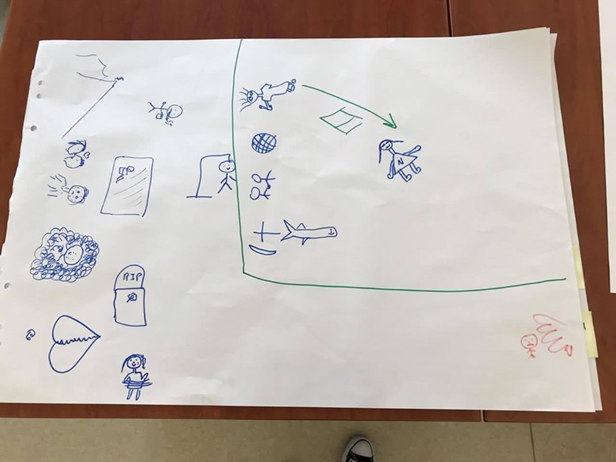


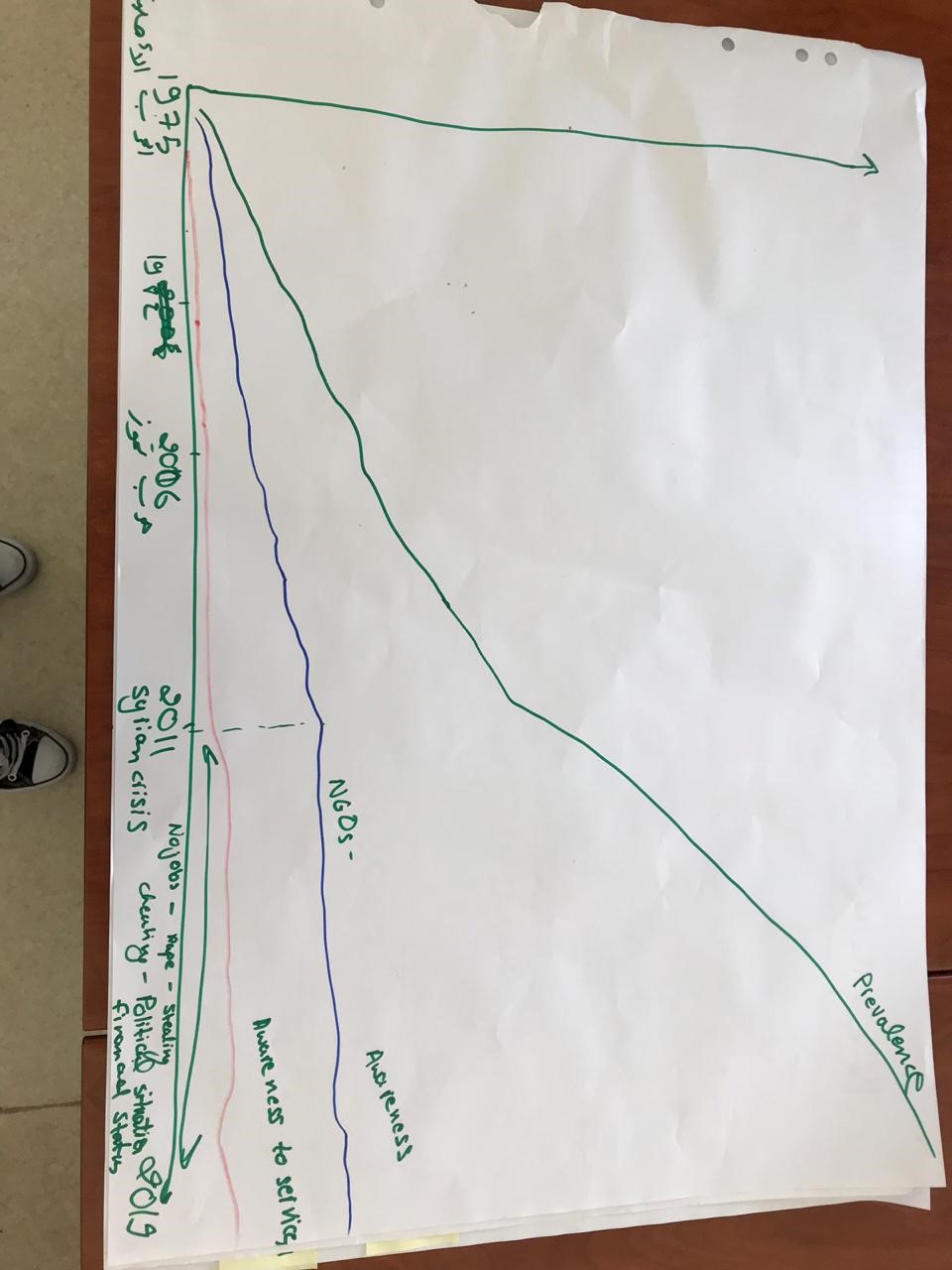


**Appendix 4**

**Table 1. Points of Fragility identified by the participants in the generated model.**

|  | **Beirut** | **Bekaa** |
| --- | --- | --- |
| **Lebanese Women** | - Finances at household - Political situation - Children’s habits - Not having support from close ones | - Financial and employment issues - Society’s perception on disability - Perpetuation of violence - Awareness to mental health help seeking |
| **Lebanese Men** | - Financial status - Political situation/ corruption - Socioeconomic status | Exercise interrupted – follow-up interviews conducted. |
| **Syrian Women** | - Discrimination - Sexual harassment for kids - Unemployment/financial situations - Rules and regulations in Lebanon | - Financial status - Violence for children - Family dynamics - Complications due to physical disabilities |
| **Syrian Men** | Exercise interrupted – follow-up interviews conducted. | - Discrimination in jobs - Rights of Syrians in Lebanon - Financial pressure - Affordability of care via health coverage |

**Table 2. Intervention suggestions by participants**

|  | **Beirut** | **Bekaa** |
| --- | --- | --- |
| **Lebanese women** | - 1. Providing job opportunities   2. Having low cost mental health services   3. Awareness campaigns on mental health issues   4. National plan for the elderly to have recreative activities   5. Emigration | - Increasing the minimum salaries - Providing health insurance - Reducing taxes - Syrians should be leaving the labour market in Lebanon Increasing awareness on mental health issues and treatment |
| **Lebanese Men** | 1. Amending taxation laws( decreasing taxes) 2. Creation of pension plans for the elderly 3. Free hospitalization policies 4. Creating job opportunities 5. Emigration 6. Awareness campaigns on mental health issues | Exercise interrupted – follow-up interviews conducted. |
| **Syrian women** | 1. Leaving to another country with better life conditions 2. Awareness campaigns for Lebanese community to accept others 3. Supervision for the humanitarian NGOs when distributing aid 4. Awareness campaigns for the humanitarian NGOs | Exercise interrupted – follow-up interviews conducted. |
| **Syrian Men** | Exercise interrupted – follow-up interviews conducted. | 1. Providing employment with better salaries 2. Improve community relations between Lebanese and Syrians 3. Raise awareness on mental health |
